# Molecular Mechanism of Sirtuin 1 Modulation by the AROS Protein

**DOI:** 10.3390/ijms232112764

**Published:** 2022-10-23

**Authors:** Sandra Weiss, Ramona S. Adolph, Kristian Schweimer, Andrea DiFonzo, Marat Meleshin, Mike Schutkowski, Clemens Steegborn

**Affiliations:** 1 Department of Biochemistry, University of Bayreuth, 95440 Bayreuth, Germany; 2 Department of Biopolymers, University of Bayreuth, 95440 Bayreuth, Germany; 3 Department of Enzymology, Charles Tanford Protein Center, Institute for Biochemistry and Biotechnology, Martin Luther University Halle-Wittenberg, 06108 Halle (Saale), Germany

**Keywords:** activator, deacetylase, Sirt1, inhibitor, regulation

## Abstract

The protein lysine deacylases of the NAD^+^-dependent Sirtuin family contribute to metabolic regulation, stress responses, and aging processes, and the human Sirtuin isoforms, Sirt1-7, are considered drug targets for aging-related diseases. The nuclear isoform Sirt1 deacetylates histones and transcription factors to regulate, e.g., metabolic adaptations and circadian mechanisms, and it is used as a therapeutic target for Huntington’s disease and psoriasis. Sirt1 is regulated through a multitude of mechanisms, including the interaction with regulatory proteins such as the inhibitors Tat and Dbc1 or the activator AROS. Here, we describe a molecular characterization of AROS and how it regulates Sirt1. We find that AROS is a partly intrinsically disordered protein (IDP) that inhibits rather than activates Sirt1. A biochemical characterization of the interaction including binding and stability assays, NMR spectroscopy, mass spectrometry, and a crystal structure of Sirtuin/AROS peptide complex reveal that AROS acts as a competitive inhibitor, through binding to the Sirt1 substrate peptide site. Our results provide molecular insights in the physiological regulation of Sirt1 by a regulator protein and suggest the peptide site as an opportunity for Sirt1-targeted drug development.

## 1. Introduction

Reversible lysine acetylation is a ubiquitous post-translational modification (PTM) regulating all types of proteins, from metabolic enzymes to scaffold and signaling proteins [1]. Sirtuins are an evolutionarily conserved family of NAD^+^-dependent protein deacetylases, with seven isoforms, Sirt1-7, in mammals [2]. They regulate metabolic programs and stress responses and have been implicated in aging-related diseases [3]. Sirtuins are part of complex signaling networks and act as metabolic sensors via their NAD^+^ dependency, and they are further regulated by PTMs and interacting proteins, but these regulatory mechanisms are little understood.

Sirt1 is a nuclear isoform and regulates, e.g., metabolism and gene expression. It is considered a therapeutic target for cancer, metabolic and neurodegenerative diseases, such as Huntington’s disease and metabolic syndrome, and a variety of small molecule Sirt1 modulators have been developed [3,4,5]. Sirt1 features the conserved Sirtuin catalytic core of ~270 amino acids, which consists of a large NAD^+^-binding Rossmann fold subdomain and a small, structurally more variable Zn^2+^-binding subdomain (Supplementary Figure S1A) [6]. The binding site for the acetyllysine-containing protein substrate is a cleft between these two subdomains, which undergo a closure movement upon binding of substrate and/or NAD^+^ cosubstrate [7,8]. The cores of the other human Sirtuin isoforms show the same fold and between 22% and 45% sequence identity to that of Sirt1, with Sirt2 and Sirt3 being its closest homologs (Supplementary Figure S1B). Sirtuins feature isoform-specific N- and C-terminal extensions, however, and the rather large Sirt1-specific extensions contribute to its stability and activation [9,10]. The C-terminal region comprises a ~25 residue “essential for Sirt1 activity” (ESA) motif, which integrates into the Rossmann fold and supports catalytic core stability and thereby activity (Supplementary Figure S1A) [10,11]. At the N-terminus, a small three-helix bundle domain mediates binding and activation by sirtuin-activating compounds (STAC) and thus is referred to as the STAC-binding domain (SBD). It appears to serve as a docking site for the compounds and to wedge them against the active site entrance of the catalytic domain/substrate complex [5,6,12].

Several interacting proteins have been described to regulate Sirt1 activity. The proteins “deleted in breast cancer 1” (DBC1) [13,14] and the human immunodeficiency virus (HIV) “transactivator of transcription” (Tat) [15] inhibit Sirt1. DBC1 appears to compete with the C-terminal ESA region for binding to the Sirt1 catalytic core to prevent Sirt1 activation by ESA [9,11]. The protein Tat was initially identified as a Sirt1 substrate, but it also binds to the Sirt1 catalytic domain in an inhibitory mode, a complex interplay that is not fully understood [15]. The only Sirt1-binding protein that seems to activate its deacetylase activity is the “active regulator of Sirt1” (AROS) [16], a small nuclear and cytoplasmic protein, which has a role in ribosomal function [17] and was originally identified as ribosomal protein S19 binding protein 1 (RPS19BP1) [18]. AROS was reported to enhance Sirt1-mediated deacetylation of its substrate p53 in vitro and in vivo, thereby inhibiting the p53-dependent transcriptional activation [16]. The Sirt1-specific activation was proposed to result from a direct interaction of AROS with Sirt1 residues 114–210, which comprise the SBD and part of the likely flexible Sirt1 N-terminus [16]. However, other studies showed activation of cellular Sirt1-dependent p53 deacetylation by AROS only after cell damaging stress [17] and in vitro even inhibition of Sirt1 by AROS instead of activation [10]. Similarly, Kokkola et al. [19] reported AROS-dependent inhibition in vitro and no Sirt1 modulation in mammalian cells after AROS overexpression and suggested that substrates, biological context, and even buffer composition might affect the interaction and the effect on SIRT1 activity.

The functional and mechanistic details of the interaction of Sirt1 and AROS thus remain to be clarified. Here, we describe a molecular characterization of AROS and how it regulates Sirt1. We find that AROS is a partly intrinsically disordered protein that inhibits through competition with the substrate peptide. Mapping interaction epitopes in Sirt1 and AROS and a crystal structure of a Sirtuin/AROS peptide complex provide a model for the regulatory complex, with an AROS motif occupying the substrate peptide binding channel. Our results thus provide molecular insights into the modulation of Sirt1 by a physiological regulator protein and suggest the peptide site as an opportunity for Sirt1-targeted drug development.

## 2. Results and Discussion

### 2.1. Molecular Characterization of Human AROS Protein

**Figure 1:**
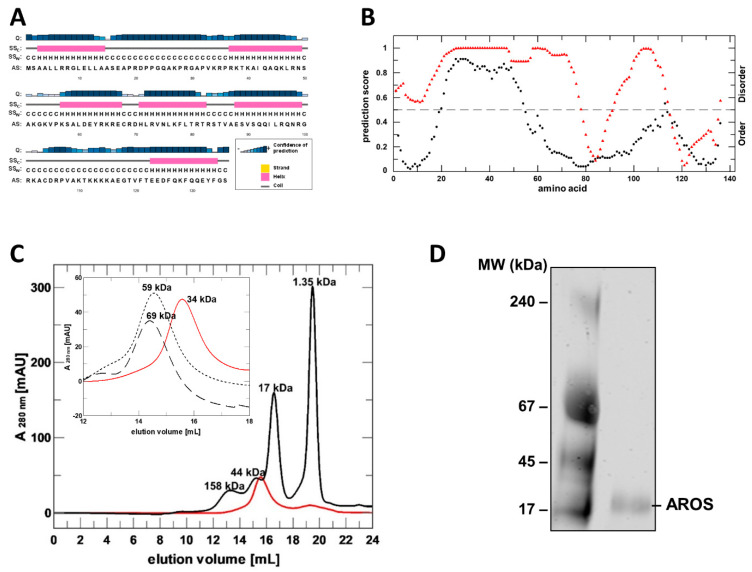
Sequence analysis, recombinant preparation and folding analysis of AROS protein. (**A**) Prediction of AROS secondary structure elements with PSIPRED (H/pink: α-helix; C/grey: coil). Confidence of prediction: blue (high), white (low). (**B**) Disorder prediction for human AROS using DISOPRED (black) and PONDR (red). (**C**) SEC elution profile of purified, refolded AROS (red) and of globular standard proteins (black; MW indicated). Inset: comparison of AROS elution profiles without additive (red) and with 4 M urea (dotted line) or 4 M GdnHCl (dashed line). MW values from comparison to standard proteins are indicated. (**D**) Blue native PAGE of refolded, purified AROS.

Analysis of the AROS protein sequence revealed no significant similarity to other protein sequences or motifs. It indicated, however, a potential disorder (Figure 1A,B). Predictions of secondary structure elements with PSIPRED [20] suggest that unstructured regions alternate with potential α-helical regions (Figure 1A). The sequences analysis with the programs DISOPRED [21] and PONDR [22] led to partially differing results (Figure 1B): For residues 21–54, DISOPRED and PONDR concordantly predicted a disordered region, while PSIPRED predicted an α-helical structure for residues 37–49. Nevertheless, these results indicate that AROS might be an intrinsically disordered protein (IDP), possibly with a partial and/or temporary fold.

For the experimental analysis of AROS’s structure and Sirt1 modulation mechanism, we aimed to establish its mg scale recombinant production in *E. coli*. AROS was highly expressed but accumulated as inclusion bodies, despite the variation of parameters such as construct and temperature. We thus established a refolding protocol for the preparation of mg amounts of AROS, starting from an earlier protocol that had yielded small amounts of soluble AROS [10]. Testing additives revealed that the addition of 0.8 M L-Arg, an established folding helper [23], and an optimized buffer and dilution kinetics enables the preparation of large amounts of soluble AROS (Supplementary Figure S2A). The final size exclusion chromatography (SEC) step yielded pure protein that eluted in a single peak and no protein in the void volume (Figure 1C; Supplementary Figure S2B), confirming that AROS assumes a soluble, non-aggregated state. This protocol now allows us to produce mg amounts of >95% pure AROS protein for functional and structural studies.

While purified AROS eluted in the size range of small, soluble proteins, the apparent molecular mass was determined to be about 30–40 kDa through comparison to elution volumes of standard proteins (Figure 1C), larger than the calculated mass of monomeric AROS (15.4 kDa AROS plus 1.9 kDa His_6_-tag). Such behavior could either indicate that AROS is a homodimer or that it has a partially unfolded structure, which extends its hydrodynamic radius compared to a globular protein and thus lets it behave like a larger globular protein in SEC. However, in SEC runs with 4 M of a chaotropic agent (urea or GdnHCl, respectively), AROS eluted at significantly higher molecular sizes (Figure 1C). This observation confirms an unfolding under denaturing conditions and thus folding-like compaction in physiological buffer conditions for at least part of the AROS protein. Our findings thus indicate that AROS either dimerizes or has a non-globular but not fully unfolded shape that causes a deviating SEC behavior. Native PAGE experiments showed a single band corresponding to the molecular mass of an AROS monomer (Figure 1D), showing that the protein is monomeric in solution and thus apparently non-globular due to partly unfolded regions.

**Figure 2:**
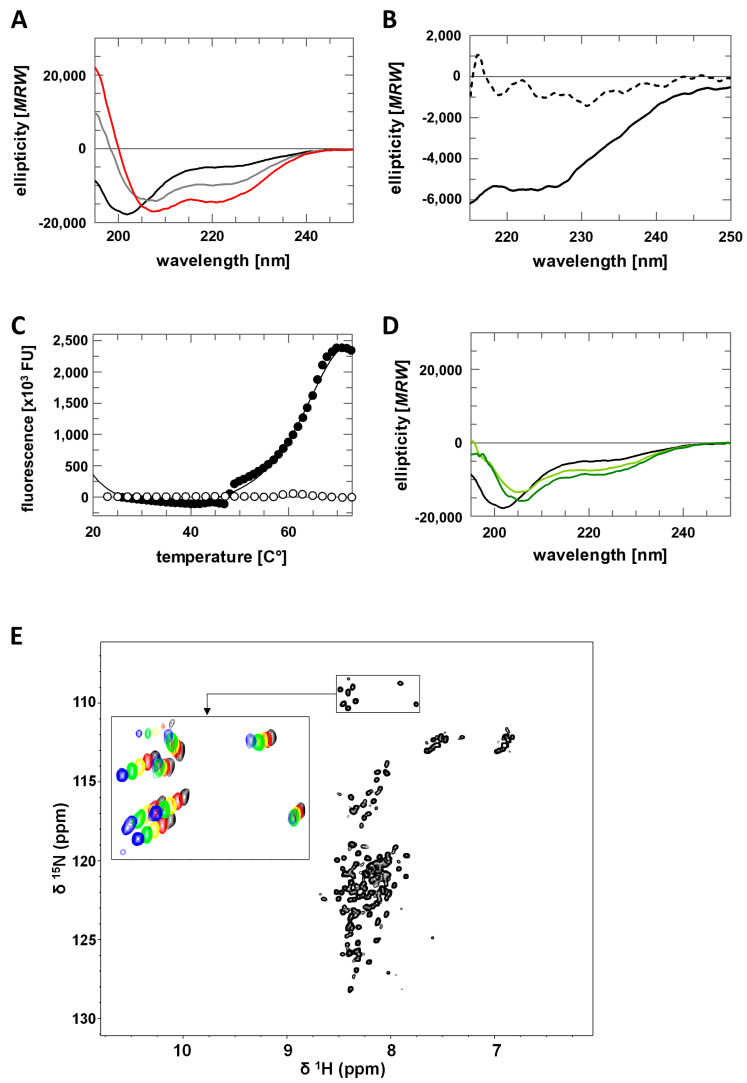
Structural characterization of AROS. (**A**) CD spectra of AROS in absence and presence of TFE (0% black, 10% grey, 20% red). (**B**) CD spectra of AROS in absence (solid line) and presence of 6 M urea (dashed line). (**C**) Thermal denaturation of AROS in absence (white) and presence of 50% TFE (black). (**D**) CD spectra of AROS in absence and presence of glycerol (0% black, 40% light green, 50% dark green). (**E**) [^1^H,^15^N]-HSQC spectra of AROS with an enlarged view of glycine signals recorded at different temperatures (25 °C black, 20 °C red, 15 °C yellow, 10 °C green, 5 °C blue).

To characterize the structure of AROS further, we first analyzed the protein through CD spectroscopy. CD spectra of AROS showed a minimum above 200 nm (203 nm) and a plateau at 217 to 227 nm (Figure 2A), which indicates the presence of α-helical structure elements. This signal could be removed by the addition of 6 M urea (Figure 2B), confirming that it results from at least a local folding of the protein. Additionally, flexible proteins or protein domains often get structured by binding of a ligand, and we thus tested for a propensity of AROS to form additional helices by adding trifluoroethanol (TFE), which can stabilize existing α-helices and induce the formation of additional ones [24,25]. Titrating AROS with TFE indeed induced increased α-helical content (Figure 2A), and with 50% TFE, a temperature-induced unfolding transition of AROS could be detected in a thermal denaturation assay (Figure 2C). AROS thus shows partial folding and a high propensity to form significant amounts of additional α-helices, which we speculate might occur under suitable conditions (such as presence of a ligand) in a physiological context. We also tested the effect of glycerol, which mimics molecular crowding effects of a physiological environment. As observed for TFE, adding glycerol to AROS induced α-helix formation (Figure 2D), identifying molecular crowding as another factor that might influence AROS folding in a physiological environment.

For a more detailed structural analysis of AROS, we then performed 2D-NMR spectroscopy. The [^1^H,^15^N]-HSQC spectrum of AROS (Figure 2E) displays poor signal dispersion in the ^1^H dimension, indicating that AROS lacks a stable and complete tertiary structure in absence of relevant factors such as an interaction partner or crowding environment. ^1^H-^15^N-HSQC spectra recorded at increasing temperatures (Figure 2E) revealed temperature-dependent signal shifts, however, showing that the AROS protein features locally folded regions, consistent with the results of the CD measurements. An attempt to determine an NMR structure of AROS nevertheless failed due to the weak dispersion, but we were successful in preparing a partial assignment of resonance for further structural studies on AROS interactions (Supplementary Table S1).

### 2.2. AROS Inhibits Sirt1 through Competition with the Acyl Substrate

**Figure 3:**
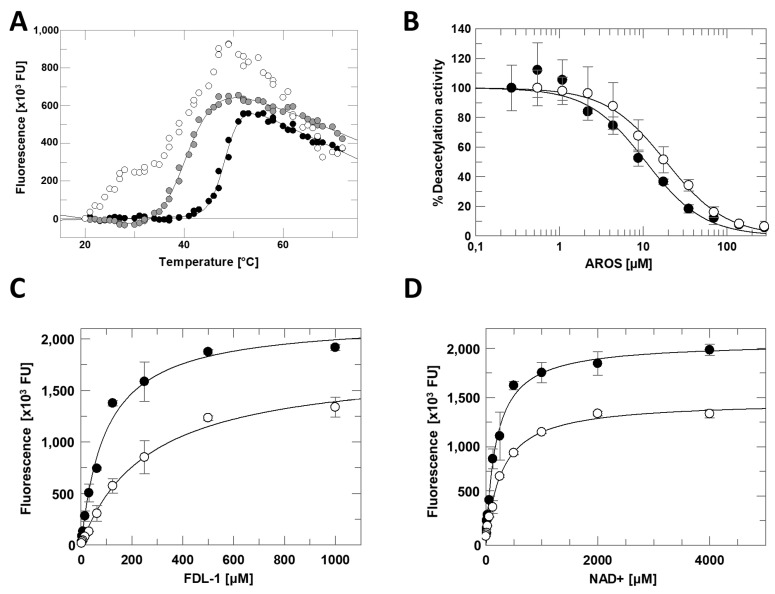
Sirt1 inhibition by AROS. (**A**) Thermal denaturation of the Sirt1/AROS complex in 20 mM Tris/HCl pH 7.0; 120 mM NaCl; 0.08% Tween 20 (white), or in 50 mM Tris/HCl pH 7.5 (grey), or in 50 mM Na_2_HPO_4_/NaH_2_PO_4_ pH 7.0 (black). (**B**) Dose-dependent inhibition of fl-Sirt1 (black) and mini-Sirt1 (white) by AROS in the FdL deacetylation assay. (*n* = 3; error bars: SD) (**C**) Substrate peptide titrations of Sirt1 in FdL activity assays in presence (white) and absence (black) of 40 µM AROS. (*n* = 3; error bars: SD) (**D**) NAD^+^ titrations of Sirt1 in FdL activity assays in presence (white) and absence (black) of 40 µM AROS. (*n* = 3; error bars: SD).

In initial attempts to analyze the regulation of Sirt1 by AROS, we observed heavy precipitation once we mixed stable solutions of the individual proteins. Therefore, we screened for buffer conditions improving the stability of the protein complex by using differential scanning fluorometry (DSF) and a 1:1 ratio of AROS and Sirt1. In a generic binding assay buffer, we observed a polyphasic transition over a broad temperature range (Figure 3A), indicating several independent unfolding events consistent with no or only weak complex formation. Under complex stabilizing conditions, an almost ideal two-state unfolding behavior is expected [26], and our screen revealed that specifically, a phosphate buffer at pH 7 displayed a significant stabilizing effect on the Sirt1/AROS complex (Figure 3A).

To analyze the functional effect of AROS on Sirt1, we performed Sirt1 activity assays using the p53-derived, fluorogenic Fluor-de-Lys (FdL) 1 substrate peptide [27]. Titrating in AROS protein caused a concentration-dependent, potent inhibition of full-length Sirt1 with an IC_50_ of 11 ± 2 µM (Figure 3B). The inhibition was confirmed in an absorption-based coupled enzymatic assay (Supplementary Figure S2C) employing a p53-derived substrate peptide devoid of FdL’s non-physiological fluorophore modification, which can artificially influence Sirtuin/ligand interactions [27]. We then analyzed the mechanism of AROS-dependent Sirt1 inhibition through competition experiments with the Sirt1 substrates, acetylated peptide, and NAD^+^. Adding 40 µM AROS caused only moderate changes in v_max_ in titrations of either substrate (Figure 3C,D). It increased K_M_ only slightly in the NAD^+^ titration, but more significantly (~3-fold) in the peptide titration, suggesting a mixed-type inhibition involving competition with the acyl substrate but not with the nucleotide cosubstrate. 

### 2.3. Mapping of the Sirt1/AROS Interaction Interfaces

Despite our successful optimization of buffer conditions for the formation of a stable Sirt1/AROS complex, we were unable to identify crystallization conditions for this complex. We, therefore, employed several alternative techniques to obtain insights into the interaction mode of the two proteins. First, we tested AROS-dependent inhibition of mini-Sirt1ex687. This shortened Sirt1 construct, comprising the core particle with SBD, catalytic domain, and ESA region, was inhibited with an IC_50_ of 19 ± 1 µM (Figure 3B), comparable to full-length Sirt1, confirming that only Sirt1′s catalytic core is relevant for the AROS interaction.

**Figure 4:**
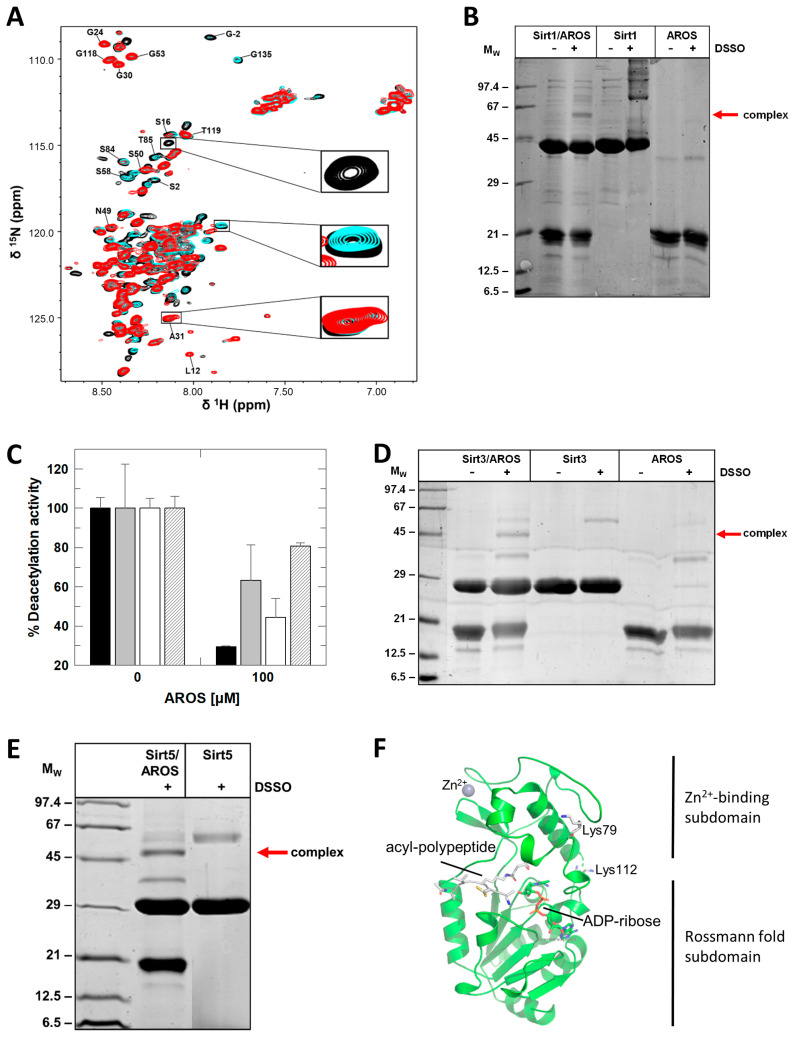
Mapping of the Sirt1/AROS interaction interfaces. (**A**) 2D [^1^H,^15^N]-HSQC spectra of the titration of 150 µM ^15^N-AROS with mini-Sirt1. Spectra corresponding to molar ratios 1:0, 1:0.5, and 1:1 are in black, cyan, and red, respectively. Examples of changed and unchanged signals are highlighted, and assigned signals are labeled (see Supplementary Figure S3 for mapping of changed signals on the sequence). (**B**) Crosslinking experiments with DSSO showing complex formation of Sirt1 with full-length AROS. (**C**) Effects of AROS on the deacetylase activities of mini-Sirt1 (black), Sirt2 (grey), and Sirt3 (white) and the desuccinylase activity of Sirt5 (striped). (*n* = 3; error bars: SD) (**D**,**E**) Crosslinking experiments with DSSO showing complex formation of AROS (**D**) with Sirt3 and (**E**) with Sirt5. (**F**) Location of Sirt5 residues identified as cross-linking sites indicated as sticks and labeled. The figure was generated from the crystal structure of a Sirt5 complex with substrate peptide and NAD^+^ analog (PDB ID 4G1C) [28].

To identify AROS regions involved in complex formation, we performed 2D-NMR titrations. Mini-Sirt1 was titrated to ^15^N-labeled AROS and 2D [^1^H,^15^N]-HSQC spectra were recorded after each step (Figure 4A). Adding Sirt1 caused significant changes in the AROS spectrum. The intensity of several signals decreased dramatically due to line broadening, indicating an intermediate chemical exchange on the NMR time scale, which is typical for complexes with μM affinity and thus consistent with our inhibition and binding data (see below). Partial assignment of AROS in the complex indicated that the very N- and C-terminal parts of AROS and the middle region from 57 to 89 are involved in binding to Sirt1 (Figure 4A; Supplementary Figure S3). We refrained from further attempts to solve a complex structure by NMR due to the pronounced AROS flexibility and considerable signal broadening observed, and the additional signal widening that would result from simulating crowding conditions to obtain a more stable complex.

To obtain additional insight, we thus analyzed the interaction strength of Sirt1 and AROS fragments using microscale thermophoresis (MST). Full-length AROS bound to mini-Sirt1ex687 with a K_d_ of 1.3 ± 0.2 μM, which is one order of magnitude below the IC_50_ and thus consistent with competition between AROS and substrate peptide. Using only the N-terminus of AROS (residues 1–89) still resulted in complex formation but increased the K_d_ ~50-fold (Table 1). Instead reducing the Sirt1 fragment to N-terminus and SBD (residues 144–230) yielded an even weaker interaction. These results indicate that N- and C-terminus of AROS contribute to binding, as well as the Sirt1 N-terminus/SBD and the catalytic core. To further map key protein regions contributing to the interaction, we also designed AROS peptides based on our NMR results and analyzed their binding to Sirt1 by using MST. The center of AROS (62–71) appears to be key to the interaction, apparently at least in part through contacts to the Sirt1-SBD, but outside regions in AROS and Sirt1 seem to contribute to their affinity (Table 2). 

To obtain further information on the Sirt1/AROS interaction interface, we performed chemical cross-linking (CL) experiments with disuccinimidyl sulfoxide (DSSO), which reacts with the Lys primary amines. DSSO treatment of a mixture of Sirtuin and AROS, but not of either protein alone, led to the formation of a potential complex band (MW of the complex: 58 kDa; Figure 4B). An attempt to identify specific linking sites by mass spectrometry failed, but consistent with our binding results that indicated binding contributions from both protein halves, CL experiments with AROS fragments truncated at the N- or C-terminus, respectively, yielded bands for the cross-linked complex comparable to each other and to full-length AROS (Supplementary Figure S4). We thus conclude that both halves of AROS contribute to binding to Sirt1, which employs its catalytic core for this interaction, supported by the SBD.

### 2.4. Structural Basis/Model for AROS-Dependent Sirt1 Modulation 

Since AROS binding is at least partially mediated by Sirt1s generic Sirtuin catalytic core, we tested the isoform specificity of AROS-dependent inhibition. Analyzing the FdL deacetylation activity of Sirt1, Sirt2, Sirt3, respective desuccinylation activity of Sirt5, in the absence and presence of 100 μM AROS, revealed that the protein inhibits all isoforms, albeit with varying potency (Figure 4C). It potently inhibits Sirt1, only weakly Sirt2 and Sirt5, and it shows a medium potency against Sirt3. An IC_50_ determination for Sirt3 yielded 48 ± 7 μM, about four times higher than for full-length Sirt1. The AROS effects on Sirt3 and Sirt5 are unlikely to be physiologically relevant due to the mitochondrial localization of these isoforms. We can conclude, however, that the key elements for Sirt1 inhibition by AROS are present in the generic Sirtuin catalytic core that is shared by all isoforms, and that we can thus exploit other isoforms as model systems for structural and mechanistic studies on its interaction with AROS. Consistently, CL experiments with Sirt3 and Sirt5 resulted in clear bands for linked complexes with AROS (Figure 4D,E). Mapping the linking sites in Sirt5 by MS identified Lys112 and Lys79, in the subdomain linker and Zn-binding module, respectively (Figure 4F).

**Figure 5:**
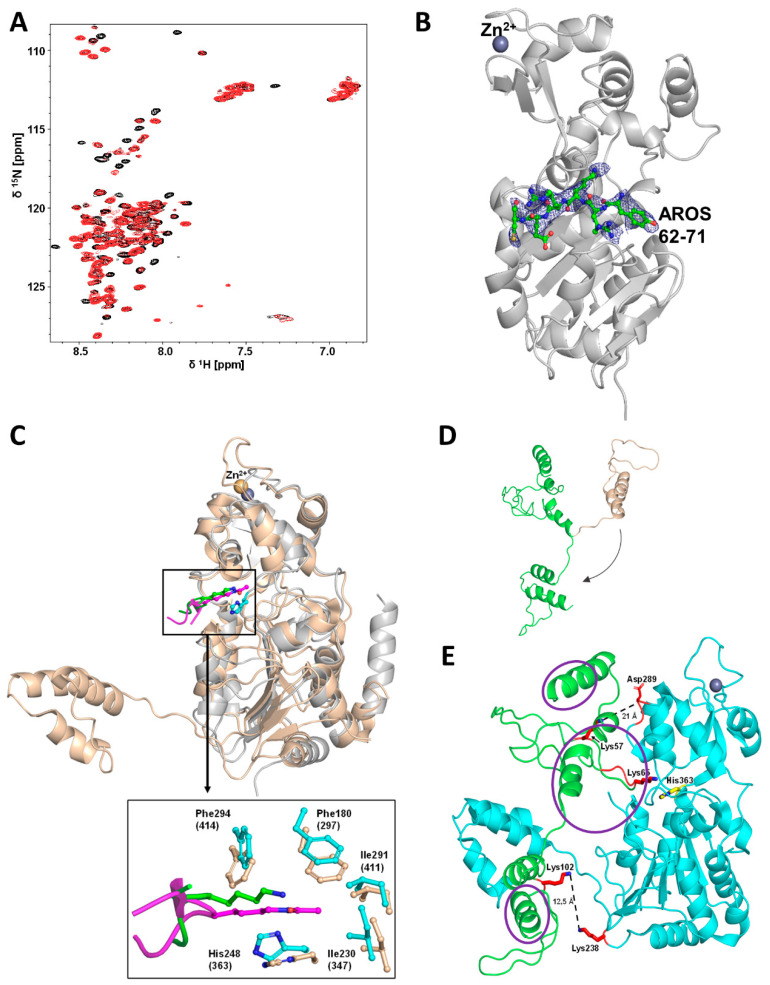
Crystal structure of a Sirt3/AROS peptide complex and modeling of the Sirt1/AROS complex. (**A**) 2D-NMR titration of ^15^N-labeled AROS with Sirt3, analogous to the experiment with Sirt1 (see Figure 4A). Shown are molar ratios 1:0 and 1:1 in black and red, respectively. (**B**) Overall structure of the complex between Sirt3 and AROS peptide 62-71 (green sticks). 2Fo-Fc electron density (blue) for AROS62-71 is contoured at 1σ. (**C**) Overlay of Sirt3 (gray cartoon) in complex with AROS62-71 (green sticks) and miniSirt1 (wheat cartoon) in complex with an acetylated p53 peptide (magenta sticks), generated through secondary structure matching for the Sirtuin cores (other overlaid Sirtuin/peptide complexes omitted for clarity). Enlarged view shows interactions of AROS62-71 and p53 with the Sirtuin active site. The Sirt3 complex is shifted slightly up compared to the Sirt1 complex for visibility. (**D**) Structure prediction for AROS using the program RaptorX (wheat cartoon). The original orientation of the C-terminus was changed manually (arrow) to obtain a more extended conformation (green cartoon) for docking onto Sirt1. (**E**) Modeling of the Sirt1 (cyan; pdb entry: 4zzh) interaction with AROS (green) based on the model in panel D, the Sirt3/AROS crystal structure, and our cross-linking results. Residues implicated in the interaction through cross-links (red) and the catalytic Sirt1 His363 (yellow) are shown as sticks, and regions implicated in the interaction through NMR are circled (purple).

To analyze the Sirtuin binding mode of AROS further, we performed 2D-NMR titrations of ^15^N-labeled AROS with Sirt3, analogous to the experiment with Sirt1. Again, adding the sirtuin resulted in significant changes in the spectrum of ^15^N-AROS (Figure 5A). The intensity of the same signals as in the Sirt1 experiment decreased, supporting our conclusion that AROS interacts with different Sirtuin isoforms equally. We thus exploited our finding that AROS inhibits also Sirt3 and Sirt5 for crystallographic studies. We solved a crystal structure of Sirt3 in complex with a peptide AROS 62-71 at 3.27 Å resolution. The structure was refined to R_cryst_/R_free_ values of 20.2/26.1% (Figure 5B; Table 3). The electron density revealed the Arg-Lys-Arg motif of the peptide ligand to be bound to the substrate binding groove, with AROS Lys65 in the active site pocket for the substrate acetyl-lysine, packed between His248 and Phe180/294 (Figure 5C). Arg66 is stacked upon Phe294, and Arg64 interacts with the mainchain oxygen of Leu298. Beyond these three AROS residues, the ligand is not well defined by electron density and appears to be flexible, without major contact with the Sirtuin. This observation is consistent with other Sirtuin/peptide crystal structures [29], which tend to feature a small, visible core around the acylated Lys but fail to show electron density for more remote residues, likely reflecting a need for local flexibility in the substrate for accessing the Sirtuin active site. Comparing the complex to other Sirtuin/peptide complexes (Sirt3/ACS2, Sirt5/IDH2, Sirt1/p53) [6,28,29] confirms that both types of ligands share the more closed Sirtuin conformation generally observed for Sirtuin/substrate complexes [7] and the high conservation of residues interacting with the visible core motif of the respective ligand (Figure 5C). The comparison indicates only subtle differences in the local Sirtuin conformation (small rotations of Sirt3 His248 and Phe294), likely caused by the Lys acylation of substrate peptides. It appears that the non-modified Lys of AROS thereby is equally well packed as an acylated Lys, consistent with the finding that acylation does not influence peptide affinity dramatically [30]. The only significant difference between the AROS motif and a substrate appears to be the side-chain position of AROS-Arg66, which appears to occupy the pocket normally accommodating the backbone of one or two additional defined residues and thereby supports AROS binding and its ability to compete with physiological substrates. This binding and inhibition mode is supported by the observed substrate peptide competition and the potency, which indicates a binding affinity to the substrate site with an affinity comparable to physiological substrates. It also rationalizes the isoform specificity of AROS: The Sirt1-specific SBD makes this isoform most sensitive (see next paragraph), and Sirt3 is most sensitive among the other isoforms because its substrate peptide binding channel shows the most pronounced negative electrostatic potential [1], complementary to the basic binding motif in AROS. 

Using our structural and mechanistic data, we then generated a model for the Sirt1/AROS complex starting from a published crystal structure of mini-Sirt1 [6] and a three-dimensional AROS model generated with the program RaptorX [31] (Figure 5D). AROS is predicted to be a flexible protein with smaller α-helical elements, consistent with our structural characterization of AROS. In a Sirt1/AROS model (Figure 5E), AROS Lys65 is positioned in the Sirt1 active site, close to the catalytic His363, as observed in our Sirt3/AROS crystal structure. Since AROS Lys102 is crosslinked with the Lys corresponding to Sirt1-Lys238, the C-terminus of unbound AROS had to be rotated relative to the AROS N-terminus to obtain a more extended AROS conformation (Figure 5D) for building a complex that rationalizes all data. This conformation allowed us to keep the crystallographically confirmed interaction of AROS-Lys65 in the substrate site and simultaneously to position AROS-Lys102 in a distance of 12.5 Å from Sirt1-238, which is consistent with DSSO cross-linking these residues. The Sirt1/AROS complex model further illustrates that AROS-Lys57 is located in the neighborhood of Asp289 of the Sirt1 zinc-binding domain (~21 Å distance), and this residue corresponds to Lys79 of Sirt5, which we found to be cross-linked to AROS-Lys57. The model thus allows us to rationalize the results of our cross-linking studies and of the structural and biochemical work characterizing the Sirtuin binding mode, such as the substrate competitive binding via the substrate polypeptide site with AROS-Lys65 mimicking a substrate site. Strikingly, the model further provides a rationale for the results of MST and NMR measurements, which indicated that AROS employs both, its N- and C-terminal parts for binding and that it can interact with the Sirtuin catalytic core as well as the Sirt1 SBD. AROS N-terminus and center occupy the Sirt1 active site and the area in front of its entrance, and the AROS C-terminus can be oriented toward the bottom of the Sirt1 core domain (Figure 5E). In this position, the Sirt1 SBD will contact the outer surface of AROS, in fact, leading to the binding of the inhibitor between the core domain and SBD in a position resembling the complex of yeast Sir2 with its regulator protein Sir4 [32]. Complex model and binding mode rationalize how the Sirt1 SBD can support AROS binding to the catalytic core and thus, why AROS can bind to several Sirtuin isoforms but shows a higher affinity for Sirt1. While an experimental structure for a Sirt1/AROS complex remains to be solved and might remain unattainable due to the technical difficulties described and discussed above, our Sirt1/AROS complex provides an excellent model for rationalizing the available experimental data and for planning future work on the regulation of Sirt1 by AROS.

## 3. Materials and Methods

### 3.1. Chemicals

If not stated otherwise, chemicals were from Sigma. Synthetic peptides were from GL Biochem (Shanghai, China; acetyl-p53: RHK-(acetyl-K)-LMFK; AROS62-71: EYRKRECRDH; AROS96-109: RQNRGRKACDRPVA; AROS1-18: MSAALLRRGLELLAASEA; AROS80-91: TRTRSTVAESVS; AROS121-134: FTEEDFQKFQQEYF). Succinylated Sirt5 FdL substrate peptide was from Enzo Life Sciences (Lörrach, Germany), and FdL1 peptide was synthesized by the coupling of Ac-R(Pbf)H(Trt)K(Boc)-OH (prepared on chlorotrityl resin) to H-Lys(Ac)-AMC x HCl in DCM-DMF (97.5:2.5, *v/v*) mixture using EDC free base/HOOBt. The product was treated with trifluoroacetic acid and purified by preparative RP-HPLC, yielding pure FdL-peptide after lyophilization.

### 3.2. Expression and Purification of Proteins

Recombinant human Sirt1, Sirt2, Sirt3, and Sirt5 were purified as described previously [6,7,33,34]. Briefly, His-tagged miniSirt1 constructs (in modified pET19b), His-Trx-tagged full-length Sirt1 (in modified pET32a), His-Trx-tagged Sirt3 residues 118–399 (in pVFT3S), His-SUMO-tagged Sirt2 residues 55–356 (in modified pET19b) and His-tagged Sirt5 residues 34–302 (in pET151/D-TOPO) were expressed in *Escherichia coli* BL21 (DE3) CodonPlus and purified through affinity chromatography with Ni-cOmplete beads (Roche). For Sirt1, Sirt3 and Sirt5, the tag was removed by incubation with Tobacco Etch Virus (TEV) protease and for Sirt2 with Senp2-protease, followed by second affinity chromatography. Sirt1 and Sirt3 were additionally purified through ion exchange chromatography. Finally, proteins were subjected to gel filtration on an S200 column (GE Healthcare), concentrated, and stored at −80 °C.

Human AROS constructs were cloned into a modified pET19b vector, resulting in N-terminal fusions to a hexahistidine tag and TEV protease cleavage site. Proteins were expressed in *E. coli* C43 (DE3) cells in LB medium with 100 μg/mL ampicillin by adding 1 mM isopropyl-β-D-thiogalactopyranoside at OD_600_ 0.8. After shaking at 37 °C for 16 h, cells were harvested, resuspended in lysis buffer (100 mM Tris/HCl pH 7; 250 mM NaCl) containing 50 µM PMSF and a protease inhibitor tablet (Roche), and sonicated on ice. Lysates were incubated with 0.5 volumes washing buffer (50 mM Tris/HCl pH 7.0, 6% (*v/v*) Triton; 1.5 M NaCl) on ice for 30 min. The inclusion bodies containing the target protein were isolated by 10 min centrifugation (4 °C, 31,000× *g*), washed twice with lysis buffer, and solubilized by sonication in 6 M GuaHCl, 100 mM Tris/HCl pH 7.0, 250 mM NaCl (5 mL for 1 g pellet) for at 20 °C for 2 h. Supernatants were separated from cell debris by centrifugation at 4500× *g* for 10 min at 4 °C, incubated with Ni-complete beads at 4 °C for 1 h, and eluted with 6 M GuaHCl, 100 mM Tris/HCl pH 7.0, 250 mM NaCl and 250 mM imidazole. Proteins were then dialyzed in 4 M GuaHCl, 100 mM Tris/HCl pH 7.0, 250 mM NaCl and renatured by diluting successively three times 50 mg protein in 1 l renaturation buffer (100 mM Tris/HCl pH 7.0, 250 mM NaCl; 0.8 M L-Arg; 5% (*v/v*) glycerol) at 4 °C and incubation for 2 h, each time. Proteins were concentrated through affinity chromatography with Ni-complete beads and renaturation buffer containing 250 mM imidazole. Eluated protein was dialyzed against 50 mM NaPO_4_ pH 7.0, 300 mM NaCl, 150 mM L-Arg, gel filtrated on a S75 column, concentrated and stored at −80 °C. 

### 3.3. Circular Dichroism (CD) Spectroscopy

CD spectra of 10 µM AROS were recorded in 10 mM NaPO4 pH 7.0 with a Jasco J-715 CD spectrometer (JASCO, Pfungstadt, Germany) at 20 °C in a 1 mm quartz cuvette and a sample volume of 200 μL. The signal was detected in the wavelength range from 250 to 195 nm, at an acquisition rate of 50 nm/min and ten accumulations.

### 3.4. Differential Scanning Fluorometry

For thermal denaturation shift assays, 2 μM AROS and 2 μM sirtuin in 50 μL buffer (thermofluor screen) were mixed with 1 μL SYPRO in a 96-well PCR plate. After adding 15 µL mineral oil, thermal denaturation by gradually increasing the temperature from 20 to 75 °C with a step size of 1 °C/min was analyzed in a FluoDiaT70 fluorescence plate reader (Otsuka Electronics, Japan). The wavelengths for excitation and emission were 465 nm and 580 nm, respectively. 

### 3.5. NMR Spectroscopy

NMR experiments were performed on Bruker Avance 700 MHz and Bruker Ascend Aeon 1000 MHz spectrometers equipped with cryogenically cooled, inverse triple resonance probes at 293 K (interaction studies) or 278 K (Resonance assignments). For interaction studies, 100 µM ^15^N-labeled AROS and 100 µM mini-Sirt1 with all components in 50 mM NaPO_4_ pH 6.5, 300 mM NaCl, and 150 mM L-Arg were used. Resonance assignments of AROS were recorded with ^13^C,^15^N-labeled protein (300 µM) in 50 mM NaPO_4_ pH 6.5, 300 mM NaCl, 150 mM L-Arg. Samples contained 10% (*v/v*) D_2_O for locking and were carried out in 3 mm tubes with an initial volume of 250 µL. NMR data were converted and processed using in-house software of the Biopolymers Department (University of Bayreuth). Two-dimensional (2D) and three-dimensional (3D) spectra were visualized and analyzed with NMRViewJ (One Moon Scientific, Inc., New York, NY, USA). 

### 3.6. Sirtuin Activity Assays

To show the influence of AROS on the activity of sirtuins the Fluor de Lys (FdL) deacylation assay with a fluorogenic peptide substrate from p53 residues 379–382 RHKK(Ac)-AMC for SIRT1–3 and an Ac-Lys-succ-AMC for SIRT5 was used. Reactions were run in a total volume of 50 μL containing 50 mM NaPO_4_ pH 7.0, 250 mM NaCl, 100 μM acetylated FdL-peptide, 500 μM NAD^+^ and 1 μM Sirtuin. After incubation at 37 °C for 30 min, reactions were stopped by adding 2 mM NAM and 10 mg/mL trypsin, incubated 20 min, and measured in a FluoDia T70 (Photon Technology, Chennai, India) at an emission wavelength of 465 nm.

The continuous coupled enzymatic assay was conducted in a total volume of 80 μL containing 50 mM Na PO_4_ pH 7.0, 250 mM NaCl, 2 mM DTT, 50 μM acetylated p53 peptide, 1 mM NAD^+^, 3.3 mM α-ketoglutarate, 37.5 U/mL GDH, 2 μM Nicotinamidase, 0.5 mM NADPH and 0.4 μM fl-Sirt1. The reactions were monitored in 30 s intervals using an Epoch 2 plate reader (BioTek, Winooski, VT, USA) at 340 nm wavelength.

All activity data shown are representatives of at least two repetitions. 

### 3.7. Chemical Crosslinking

The lysine-reactive homofunctional crosslinkers disuccinimidyl sulfoxide (DSSO) and glutaraldehyde were used for crosslinking of proteins. Reactions were performed in 50 mM NaHPO4 pH 7, 250 mM NaCl with 50 µM of each protein, with all samples dialyzed in Xpress Micro Dialyzers (MWCO: 6 kDa, Serva) before the reaction. An amount of 2 mM DSSO or 0.22 mM glutaraldehyde were added, reactions incubated at RT, and 1/10 of the reaction volume taken at different times and supplemented with 1/2 volume stop solution (1 M Tris/HCl pH 8). Samples were mixed with SDS sample buffer (50 mM Tris/HCl pH 6.8, 2% (*w/v*) SDS, 100 mM β-mercaptoethanol, 0.02% (*w/v*) bromophenol blue), incubated for 5 min at 95 °C and analyzed by SDS-PAGE. Bands were cut from gels and trypsinized as described by [35]. LC- ESI-MS/MS of 5 µL of each sample was performed on an LTQ-XL mass spectrometer coupled with EASY-nLC II chromatographic system (ThermoScientific, Waltham, MA, USA) using a self-packed column with ReproSil-Pur C18-AQ 3 µm beads (Dr. Maisch GmbH, Ammerbuch, Germany). Protein identification was completed with the program Byonic (Protein Metrics, Cupertino, CA, USA).

### 3.8. Binding Measurements

Dissociation constants (K_d_) were determined using microscale thermophoresis (MST). For the MST measurements, either AROS or Sirt1 were initially labelled with a fluorophore. For the coupling reaction, 50 μM of the respective protein was incubated with 50 μM Cy5-NHS ester, which reacts with lysines on the surface of the protein, for one hour in the dark in 50 mM Na_2_HPO_4_/NaH_2_PO_4_ pH 8.0 at RT, followed by removal of non-bound Cy5 by NAP5 column (GE Healthcare, Chicago, IL, USA). The measurement was performed in 50 mM Na_2_HPO_4_/NaH_2_PO_4_ pH7.0, 250 mM NaCl and 2 mg/mL BSA, at 20 °C and an absorption wavelength of 630 nm and emission wavelength of 670 nm. A monolith NT.115 (Nanotemper Technologies, LED power 50–70%, laser power 50–70%) was used, and 40–120 nM fluorescently labelled protein. Measurements and evaluation were performed with the Nanotemper MO software v2.2.4. 

### 3.9. Crystallization and Structure Determination of Sirtuin Complexes 

Sirt3/AROS-Peptide complexes were crystallized in 0.4 μL sitting drops at 20 °C. An amount of 10 mg/mL Sirt3 were pre-incubated with 1 mM AROS peptide 62–71 and 1 mM AROS peptide 96–109 for 16 h at 20 °C and then centrifuged at 15,000× *g* for 10 min. Crystals grew with the reservoir condition 12% PEG 8.000, 0.1 M HEPES pH 7.5, and 0.2 M NaCl from the Protein Complex Suite screen (Quiagen, Hilden, Germany). Crystals were washed in cryoprotectant consisting of reservoir solution with 25% (*v/v*) glycerol and 1 mM of the AROS peptides and shock frozen in liquid nitrogen. Diffraction data were collected at 100 K on beamline 14.1 of BESSY II, operated by Helmholtz-Zentrum Berlin (HZB; Berlin-Adlershof, Germany) [36]. Diffraction data were processed using XDSapp [37] and structures were solved by “molecular replacement” phasing with the software Phaser [38] and using PDB entry 3GLS [29] as the search model. The structure was rebuilt in Coot [39] and refined with Phenix [40]. 

## Figures and Tables

**Table 1:** K_d_ of Sirt1 interactions with AROS constructs.

Sirt1 Construct	AROS Construct	K_d_ [µM]
miniSirt1ex687	AROS 1–136	1.3 ± 0.2
miniSirt1ex687	AROS 1–89	65 ± 9
Sirt1 144–230	AROS 1–89	209 ± 67

**Table 2:** K_d_ of Sirt1 interactions with AROS peptides.

AROS Peptide	Sirt1 Construct	K_d_ [µM]
AROS 62–71	miniSirt1ex687	86 ± 16
AROS 62–71	Sirt1 144–230	166 ± 24
AROS 80–91	miniSirt1ex687	721 ± 310
AROS 80–91	Sirt1 144–230	579 ± 102
AROS 96–109	miniSirt1ex687	151 ± 31

**Table 3:** Diffraction data and refinement statistics.

Data Collection		
Space group		P3_2_21
Cell dimensions		a = b = 110.2 Å; c = 344.4 Å
Resolution (Å) ^a^		49.19–3.27 (3.38–3.27)
R_merge_^a^		0.316 (3.191)
CC_1/2_^a^ (%)		99.7 (33.9)
I/σI ^a^		7.9 (0.7)
Unique reflections		38,569
Completeness (%) ^a^		99.7 (98.7)
Redundancy ^a^		10.9 (10.8)
**Refinement**		
Resolution (Å)		49.19–3.27
No. reflections		38,557
R_work_/R_free_ (%)		20.2/26.1
No. atoms	Protein	13,131
B-Factors (Å^2^)	Protein	95.5
R.m.s. deviations	Bond lengths (Å)	0.01
	Bond angles (°)	1.4

^a^ Highest resolution shell is shown in parentheses.

## Data Availability

Coordinates and structure factors for the Sirt3/AROS peptide complex have been deposited with the Protein Data Bank (www.rcsb.org) under accession code 8BBK.

## Supplementary Materials

The following supporting information can be downloaded at: https://www.mdpi.com/article/10.3390/ijms232112764/s1.
