# Supplementary material for: Molecular Mechanism of Sirtuin 1 Modulation by the AROS Protein
Source: Int J Mol Sci. 2022 Oct 23;23(21):12764. doi: 10.3390/ijms232112764 (PMC9654219; doi:10.3390/ijms232112764)
Supplement: Supplementary file 1 [file ijms-23-12764-s001.zip › ijms-1954003-supplementary.pdf]

**Supplementary Materials**

(**A**).

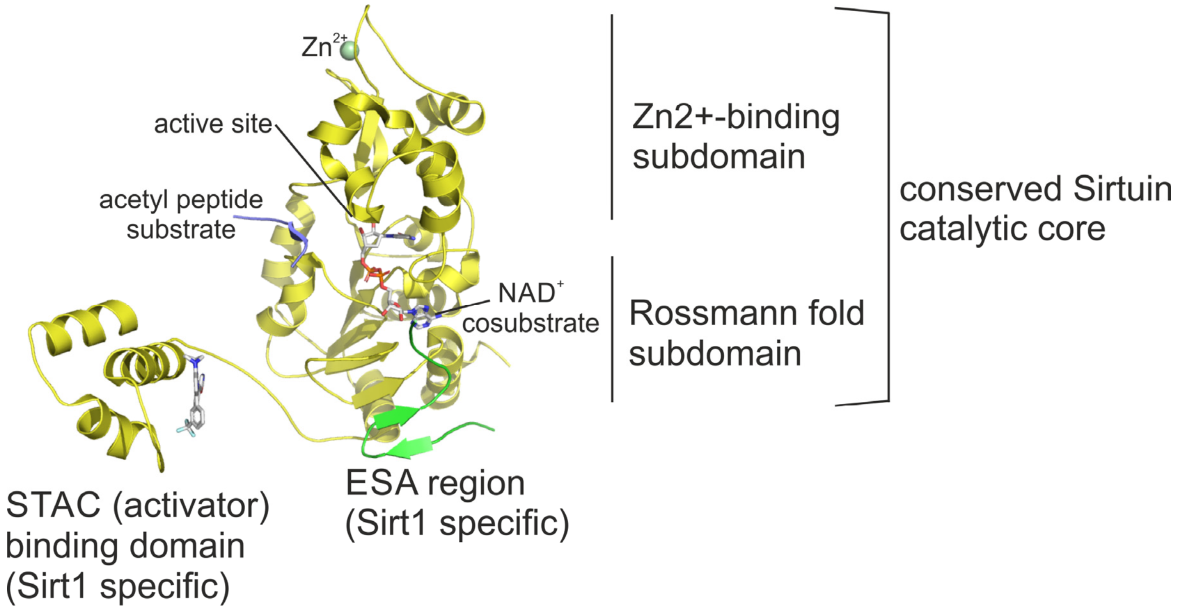


(**B**).

Percent Identity Matrix

| | | | | | | | |
|---|---|---|---|---|---|---|---|
| hSirt1 | 100.00% | 44.84% | 41.83% | 25.42% | 28.69% | 21.59% | 22.91% |
| hSirt2 | 44.84% | 100.00% | 54.02% | 24.18% | 25.73% | 25.32% | 23.61% |
| hSirt3 | 41.83% | 54.02% | 100.00% | 26.14% | 28.15% | 29.57% | 26.52% |
| hSirt4 | 25.42% | 24.18% | 26.14% | 100.00% | 27.03% | 29.18% | 29.13% |
| hSirt5 | 28.69% | 25.73% | 28.15% | 27.03% | 100.00% | 23.25% | 25.44% |
| hSirt6 | 21.59% | 25.32% | 29.57% | 29.18% | 23.25% | 100.00% | 40.83% |
| hSirt7 | 22.91% | 23.61% | 26.52% | 29.13% | 25.44% | 40.83% | 100.00% |

**Figure S1. Overall structure of human Sirt1 and conservation of the catalytic core.** (**A**) The conserved Sirtuin catalytic core and the Sirt1-specific ESA region and STAC-binding domain (with bound activator as sticks) are indicated. The acetyl substrate is show in blue, and the NAD$^+$ cosubstrate as sticks colored according to atom type. (**B**) Identities among human Sirtuin catalytic cores. hSirt1 (Uniprot Q96EB6): residues 240-498; hSirt2 (Q8IXJ6): 61-340; hSirt3 (Q9NTG7): 122-382; hSirt4 (Q9Y6E7): 41-312; hSirt5 (Q9NXA8): 37-301; hSirt6 (Q8N6T7): 31-274; hSirt7 (Q9NRC8): 86-330.

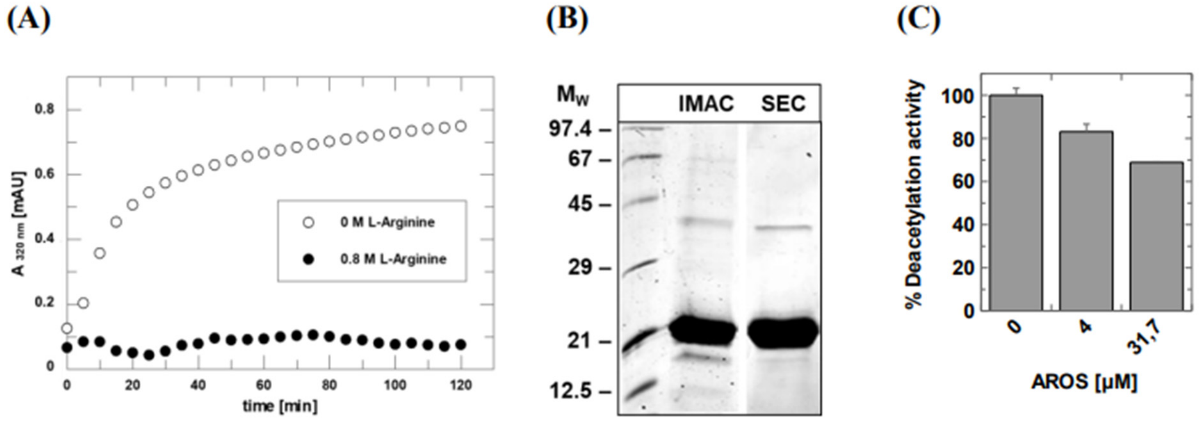


**Figure S2. AROS refolding and AROS-dependent Sirt1 inhibition.** (**A**) Monitoring of aggregation during AROS refolding experiments in presence and absence of L-Arg through absorption measurements at 320 nm. (**B**) SDS-PAGE analysis of AROS purity after second affinity chromatography (IMAC) and SEC step. (**C**) Inhibition of Sirt1 by refolded AROS measured in a coupled enzymatic deacetylation assay.

```
    -10        1          10          20         30         40
MGHHHHHHAENLYFQGH MSAALLRRGL ELLAASEAPR DPPGQAKPRG APVKRPRKTK

         50         60         70         80         90        100
AIQAQKLRNS AKGKVPKSAL DEYRKRECRD HLRVNLKFLT RTRSTVAESV SQQILRQNRG

        110        120        130
RKACDRPVAK TKKKKAEGTV FTEEDFQKFQ QEYFGS
```

**Figure S3. Amino acid sequence of 6xHis-TEV-AROS and residues interacting with Sirt1.** The sequence of the complete AROS protein construct used in interaction experiments, including His-tag and TEV protease cleavage site, is shown with numbering. Residues contributing to the interaction with Sirt1 are coloured red, and residues not involved in the interaction are coloured cyan. .

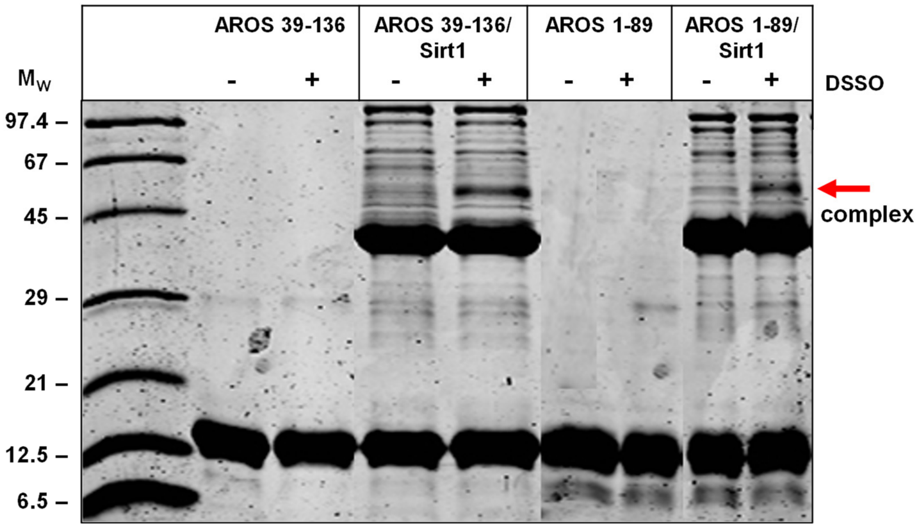


**Figure S4. Cross-linking of AROS fragments with Sirt1:** AROS fragments representing N- or C-terminus of the protein, respectively, were cross-linked with Sirt1 using DSSO.

**Table S1.** Partial assignment of NMR resonance for 6xHis-TEV-AROS protein.

| residue[a] | δ $^{1}$H [ppm] | δ $^{13}C_{\alpha}$ [ppm] | δ $^{13}C_{\beta}$ [ppm] | δ $^{15}N_{\beta}$ [ppm] |
|---|---|---|---|---|
| (Q)G-2 | 8.002 | 45.3742 | - | 108.7976 |
| (M)S2 | 8.3802 | 58.559 | 63.833 | 115.103 |
| (E)L12 | 8.168 | 56.36 | 42.15 | 127.22 |
| (A)S16 | 8.2232 | 58.7974 | - | 114.6421 |
| (P)G24 | 8.6448 | 45.3724 | - | 109.4523 |
| (R)G30 | 8.5561 | 44.8977 | - | 110.6427 |
| (G)A31 | 8.302 | 50.54 | 18.09 | 125.25 |
| (I)Q43 | 8.564 | 56.296 | 29.58 | 124.92 |
| (R)N49 | 8.589 | 53.58 | 38.65 | 119.99 |
| (N)S50 | 8.394 | 63.76 | 58.67 | 116.739 |
| (K)G53 | 8.4771 | 45.2154 | - | 110.1696 |
| (G)K54 | 8.262 | 56.13 | 33.05 | 121.182 |
| (K)S58 | 8.574 | 58.55 | - | 116.4649 |
| (R)S84 | 8.526 | 58.447 | - | 117.1726 |
| (S)T85 | 8.3557 | 62.0539 | - | 116.036 |
| (E)G118 | 8.5994 | 45.2948 | - | 110.325 |
| (G)T119 | 8.1692 | 62.059 | 68.963 | 114.7172 |
| (F)G135 | 7.796 | 45.407 | - | 110.259 |
| G | 8.5615 | 45.247 | - | 108.689 |
| G | 8.4133 | 46.321 | - | 108.7166 |
| G | 8.619 | 45.2948 | - | 110.439 |
| G | 8.4736 | - | - | 109.2302 |
| T | 8.1569 | 62.059 | - | 114.3915 |

[a] Numbering for human 6xHis-TEV-AROS protein (see Suppl. Fig S3). When residues are listed without number, only the type of amino acid could be assigned. The residue in parentheses indicates the type of amino acid preceding this residue, which was utilized for assignment.
